# *OsMPK6* plays a critical role in cell differentiation during early embryogenesis in *Oryza sativa*

**DOI:** 10.1093/jxb/erw052

**Published:** 2016-02-24

**Authors:** Jakyung Yi, Yang-Seok Lee, Dong-Yeon Lee, Man-Ho Cho, Jong-Seong Jeon, Gynheung An

**Affiliations:** Crop Biotech Institute and Graduate School of Biotechnology, Kyung Hee University, Yongin, 446–701, Republic of Korea

**Keywords:** Axis formation, embryogenesis, globular embryo, OsMPK6, phytoalexin, rice.

## Abstract

The formation of body axes is the basis of morphogenesis during plant embryogenesis. We identified embryo-lethal mutants of rice (*Oryza sativa*) in which T-DNAs were inserted in *OsMPK6*. Embryonic organs were absent because their development was arrested at the globular stage. Similar to observations made with *gle4*, *shootless*, and *organless*, the *osmpk6* mutations affected the initial step of cell differentiation. Expression of an apical–basal axis marker gene, *OSH1*, was reduced in the mutant embryos while that of the radial axes marker genes *OsSCR* and *OsPNH1* was not detected. The signal for *ROC1*, a protodermal cell marker, was weak at the globular stage and gradually disappeared. Transcript levels of auxin and gibberellin biosynthesis genes were diminished in *osmpk6* embryos. In addition, phytoalexin biosynthesis genes were down-regulated in *osmpk6* and a major diterpene phytoalexin, momilactone A, did not accumulate in the mutant embryos. These results indicate that *OsMPK6* begins to play a critical role during early embryogenesis, especially when the L1 radial axis is being formed.

## Highlight

The MAP kinase cascade controls early embryogenesis prior to axis formation.

## Introduction

Improving our understanding of the molecular mechanisms and cellular events involved in embryogenesis could help us increase grain yields and seed quality. During the early stage of that biological process, the apical–basal axis and the radial axis, perpendicular to it, are the first to be generated. The shoot apical meristem (SAM), cotyledon, hypocotyl, and root apical meristem are then formed along the apical–basal axis, while the epidermis (L1), ground tissue (L2), and central vascular cylinder (L3) are concentrically arranged from outside to inside along the radial axis (Mayer *et al.*, 1991). Monocotyledon plants such as rice (*Oryza sativa*) and maize (*Zea mays*) have another axis, the dorsal–ventral axis, and their embryos are not radially symmetrical. There, shoot development is defined on the ventral side, opposite to the dorsal side (Sato, 2008). In *Arabidopsis*, the genetic control of pattern formation and organ differentiation has been elucidated by analyses of embryonic mutants and marker gene expression (Jenik *et al.*, 2007; ten Hove *et al.*, 2015).

Despite these advancements, little is known about the molecular mechanisms for rice embryogenesis. More than 200 rice embryo mutants have been categorized into phenotypic groups (Hong *et al.*, 1995). Lethal mutant classifications include *embryoless*, *organless*, *shootless* (*shl*), and *radicleless*. Seeds of the *embryoless* mutant have only an endosperm, whereas *organless*, *shl*, and *radicleless* mutants are defective in embryonic organs, shoots, and radicles, respectively. All are characterized by defects during early embryonic development or cell differentiation. The *organless1* mutants fail to develop most organs and tissues, although the scutellum and endosperm are normal (Hong *et al.*, 1995). Another mutant, *tryptophan deficient dwarf* (*tdd1*), is defective in a gene that encodes a protein homologous to anthranilate synthase β-subunit, which catalyses the first step of the Trp biosynthesis pathway (Sazuka *et al.*, 2009). Because indole acetic acid (IAA) is biosynthesized from Trp, the phenotype is probably due to deficiencies of Trp and IAA.

During embryogenesis the SAM develops first and then serves as the basis for the production of aboveground plant parts, including the leaves, stems, and axial buds (Steeves and Sussex, 1989). In rice, the *shl* mutants fail to develop the SAM (Satoh *et al.*, 1999), while *shoot organization* (*sho*) mutants have a large, flattened shoot apex (Hong *et al.*, 1995). Among these mutants, weak *shl* alleles show a phenotype similar to that of *sho* mutants, that is, an increased rate of leaf production and random phyllotaxy (Itoh *et al.*, 2000). This suggests that *SHL* and *SHO* genes function in both the initiation and the maintenance of the SAM (Itoh *et al.*, 2000; Satoh *et al.*, 2003). Expression of two Class III homeodomain leucine zipper (HD-ZIPIII) genes, *OSHB1* and *OSHB2*, is decreased in *shl2*, *shl4*/s*ho2*, and *sho1* mutants, whereas transcript levels of *miRNA166* are increased in those mutants. Both *OSHB1* and *OSHB2* have a *miR166* recognition sequence (Nagasaki *et al.*, 2007), indicating that small RNA acts as a signalling molecule to induce SAM initiation. Two other HD-ZIPIII genes, *OSHB3* and *OSHB4*, are also expressed in the central and ventral domains of an embryo, where the SAM is later initiated (Itoh *et al.*, 2008).

Mitogen-activated protein (MAP) kinase cascades consist of kinase signalling modules that are evolutionarily conserved throughout eukaryotes (Ichimura *et al.*, 2002). The cascade is a hierarchical organization of three classes of functionally related kinases: MAP kinase (MPK), MPK kinase (MAP2K or MKK), and MAP2K kinase (MAP3K or MEKK). This cascade amplifies and integrates the signals between the cellular environment and metabolic/transcriptional responses (Hamel *et al.*, 2006). *OsMPK6* (previously referred to as *OsMPK2* by Kurusu *et al.*, 2005; *OsMAPK6* by Lieberherr *et al.*, 2005; *OsSIPK* by Lee *et al.*, 2008; and *OsMPK1* by Singh *et al.*, 2012) was isolated from a suspension cell culture treated with a sphingolipid elicitor (SE) that was purified from the rice blast fungus (*Magnaporthe oryzae*) (Lieberherr *et al.*, 2005). After SE treatment, OsMPK6 protein and mRNA levels remained unchanged. However, kinase activity of the protein was rapidly induced in response to the elicitor in rice cells. Post-translational activation of OsMPK6 was also observed in *OsMKK4*-overexpressing cells treated with a chitin elicitor. This indicates that OsMKK4 is an upstream kinase for OsMPK6 and that the OsMKK4–OsMPK6 cascade is activated by the elicitor (Kishi-Kaboshi *et al.*, 2010).

Phytoalexins are low-molecular-weight antimicrobial compounds produced in response to pathogens (Tsuji *et al.*, 1992). Rice plants accumulate diterpenoid phytoalexins, momilactones, and phytocassanes upon *M. oryzae* infection (Cartwright *et al.*, 1981; Peters, 2006). Phytoalexin levels are increased in cells expressing a constitutively active form of *OsMKK4*. This demonstrates that the elicitor-responsive OsMKK4–OsMPK6 cascade is essential for phytoalexin biosynthesis (Kishi-Kaboshi *et al.*, 2010). *Arabidopsis* MPK6 and MPK3 belong to the same clade as rice OsMPK6 in the MPK family. The *Arabidopsis* proteins function together in a single MAP kinase cascade and are involved in various signalling responses. The MKK4–MPK3/MPK6 cascade leads to phytoalexin biosynthesis upon pathogen infection (Ren *et al.*, 2008). MPK6 activated by *Botrytis cinerea* phosphorylates WRKY33, which then induces biosynthesis of camalexin, the major phytoalexin in *Arabidopsis* (Mao *et al.*, 2011).

In addition to the activation of defence genes, MAP kinases regulate stomatal patterning in leaves (Wang *et al.*, 2007), ethylene production by phosphorylating a subset of ACC synthase isoforms (Liu and Zhang, 2004; Joo *et al.*, 2008; Han *et al.*, 2010), generation of reactive oxygen species, and hypersensitive responses such as cell death (Ren *et al.*, 2002; Kim and Zhang, 2004; Liu *et al.*, 2007; Lee, 2014). They are also involved in inflorescence architecture (Bush and Krysan, 2007; Meng *et al.*, 2012), anther and embryo development (Bush and Krysan, 2007; Wang *et al.*, 2007), and seed formation and root development (López-Bucio *et al.*, 2014). Here, we report that *OsMPK6* is essential for embryogenesis in rice.

## Materials and methods

### Genotyping and identification of *OsMPK6*/*osmpk6*

Seeds from heterozygote rice lines 3A-60391 (*OsMPK6/osmpk6-1*) and 2A-10337 (*OsMPK6/osmpk6-2*) were soaked in water for 12h. After imbibition, embryos were extracted with a razor blade. DNA was isolated by the hexadecyltrimethylammoniumbromide method (CTAB; Chen and Ronald, 1999). Genotypes were determined by PCR, using gene-specific primers and T-DNA primers (see Supplementary Table S1 at *JXB* online).

### RNA extraction and quantitative reverse transcription PCR

Developing embryos were isolated under a microscope after briefly dipping seeds collected at 6 days after pollination (DAP) in liquid nitrogen. Their endosperms were separated under a microscope to avoid pericarp contamination. Total RNA was isolated and cDNAs were synthesized as previously reported (Lee and An, 2015). Gene expression was monitored by quantitative reverse transcription (qRT)-PCR as described previously (Lee and An, 2015). All experiments were conducted at least three times, using three or more independent samples per experiment. Primers for analysing transcript levels are listed in Supplementary Table S1.

### Histochemical analyses

Developing seeds at various stages after pollination were fixed with 3% (w/v) paraformaldehyde and dehydrated in an ethanol series as previously described (Yi *et al.*, 2012). The samples were embedded in Paraplast, then sectioned to an 8 μm thickness with a rotary microtome. After staining with 0.05% toluidine blue, they were observed with an Olympus microscope BX61 (Olympus, http://www.olympus.com).

### RNA *in situ* hybridization

Developing seeds were fixed in 0.05M sodium phosphate buffer (pH 7.2) containing 4% paraformaldehyde and 0.25% glutaraldehyde. Samples were dehydrated, embedded, sliced, and attached to slides as previously described (Lee *et al.*, 2007). For preparation of digoxigenin-labelled RNA probes, the coding regions were PCR-amplified. Primers used for the probes are listed in Supplementary Table S1. The PCR products were cloned into p-GEM-T vectors (Promega, Madison, WI, USA), then linearized and used as templates for making the digoxigenin-labelled sense and antisense RNA probes. Tissue sections were cleared with xylene and dehydrated through a graded ethanol series. After they were hybridized with the labelled RNA probe, the sections were incubated overnight at 60°C and washed with 0.15M sodium chloride and 15mM sodium citrate. The labelled probes were detected with anti-digoxigenin alkaline phosphatase (Roche, Mannheim, Germany) in combination with nitro blue tetrazolium chloride/5-bromo-4-chloro-3 indolyl phosphate p-toluidine salt (Roche).

### Measurement of phytoalexin content

Wild-type (WT) and *osmpk6-1* seeds were imbibed in distilled water for 48h at 28ºC, and subsequently treated for 72h at 28ºC with elicitors—115 µg mL^−1^ flg22 peptide (AnaSpec, Fremont, CA, USA) and 60 µg mL^−1^ hexa-*N*-acetylchitohexaose (Megazyme, Wicklow, Ireland)—or water as the mock control. After treatment, embryos (approximately 0.15g) were separated and collected. The samples were pulverized in liquid nitrogen and extracted overnight with ethyl acetate by stirring. After centrifugation, the ethyl acetate extract was dried under vacuum. The residue was dissolved in methanol and used in HPLC-electrospray ionization-MS/MS analysis to measure levels of phytoalexin momilactone A in the embryo samples as described previously, with a minor modification (Shimizu *et al.*, 2008). Standard momilactone A was kindly provided by Dr Morifumi Hasegawa (Ibaraki University, Japan). Each sample was analysed using a reversed-phase HPLC equipped with a ZORBAX Eclipse Plus C_18_ column (2.1mm in diameter, 100mm long; Agilent Technologies, Santa Clara, CA, USA) in MeCN, H_2_O, and AcOH (70.0:29.9:0.1, v:v:v) at a flow rate of 0.2mL min^−1^. The eluent was monitored by a triple quadrupole mass spectrometer (Agilent 6410B; Agilent Technologies) fitted with an electrospray ion source in positive ion mode. The electrospray capillary was 4kV and the source temperature was 350ºC. The precursor and product ions were monitored by the MRM mode to determine momilactone A contents.

## Results

### Mutations in *OsMPK6* cause embryo-lethal phenotypes

**Fig. 1.**
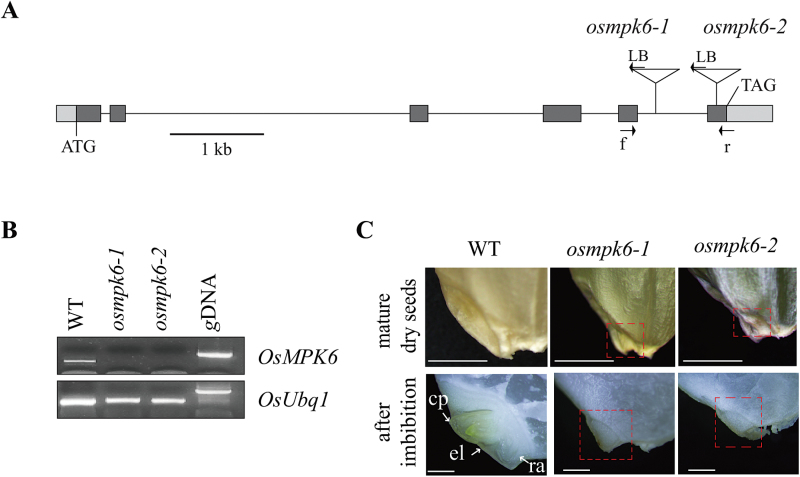
Characterization of T-DNA insertion mutants in *OsMPK6.*

To identify genes essential for zygote development, we analysed the genotype data from our T-DNA mutant pool (An *et al.*, 2003; Jeon *et al.*, 2000; Jeong *et al.*, 2002). We selected lines with a segregation ratio close to 1:2:0 (WT to heterozygote to homozygote). Among them, we studied lines 3A-60391 and 2A-10337, in which T-DNA is inserted into the fifth intron and the sixth exon of *OsMPK6,* respectively (Fig. 1A). Tests of seeds from the heterozygous parent showed that 19.8% (18/91) of *osmpk6-1* and 20.75% (22/106) of *osmpk6-2* did not germinate (Table 1). The ratio between germinated and non-germinated seeds was approximately 3:1. Genotyping revealed that all of the non-germinated seeds were homozygotes while those that germinated were WT or heterozygotes, thereby indicating that the T-DNA insertion caused the lethality. Furthermore, transcripts of *OsMPK6* were not detected in the non-germinated seeds, suggesting that both mutations are null alleles (Fig. 1B). When compared with the WT, mutant seeds had small, flat embryos (Fig. 1C). After 12h of imbibition, embryonic organs, including the coleoptile, first leaf, and radicle, appeared from the WT but not from the mutant (Fig. 1C). To examine whether overexpression of *OsMPK6* affects embryo morphology, we identified an activation-tagging line (*OsMPK6-D*, 2A-10648) in which the *35S* enhancer elements are located 2124bp downstream from the stop codon of *OsMPK6* (Supplementary Fig. S1A). In that line, transcript levels were significantly enhanced (Supplementary Fig. S1B), but no obvious phenotypic changes were observed in plants, including the embryos (Supplementary Fig. S1C).

**Table 1.** Segregation analysis of *OsMPK6/osmpk6-1* and *OsMPK6/osmpk6-2*

	Germinated seeds (G)		Non-germinated seeds (N)	Observed ratio (G:N)	Expected ratio (G:N)	χ^2^
	WT	heterozygote	homozygote			
*OsMPK6/osmpk6-1* (n = 91)	23	50	18	4.06:1	3:1	1.322 (*P*<0.05)
*OsMPK6/osmpk6-2* (n = 106)	27	57	22	3.82: 1	3: 1	1.019 (*P*<0.05)

The segregation ratio was analysed in progeny of selfed *OsMPK6/osmpk6* mutant lines. χ^2^ tests were performed to evaluate the relationship between the observed data and the predicted ratio of 3:1.

(**A**) Gene structure of *OsMPK6*. Dark-grey boxes indicate exons; light-grey boxes, UTRs; lines connecting boxes, introns. Triangles are T-DNA insertions of *osmpk6-1* (line 3A-60391) and *osmpk6-2* (line 2A-10337). Arrows f (forward), r (reverse), and LB (left border) are primers used for genotyping and RT-PCR analyses of *OsMPK6* transcript levels. Scale bar = 1kb. (**B**) Measurement of *MPK6* transcript levels by RT-PCR. Rice *Ubiquitin 1* (*OsUbi1*, Os03g0234200) served as control. (**C**) Embryo phenotypes of WT, *osmpk6-1*, and *osmpk6-2*. Upper panel presents mature embryos from dry seeds; lower panel, embryos after imbibition. Red dotted boxes show the mutant embryo regions. cp, coleoptile; el, embryonic leaves; ra, radicle. Bars = 1mm. (This figure is available in colour at *JXB* online.)

As a member of the MPK family, *OsMPK6* encodes a 398-residue protein that contains Thr-Glu-Tyr residues within the phosphorylation-activation motif (Supplementary Fig. S2A). Among the 20 MPK members in *Arabidopsis*, OsMPK6 was the most homologous to MPK6, with 85% identity (Supplementary Fig. S2A, B). This protein also had a predicted docking groove for binding substrates as well as the 11 conserved protein kinase domains and conserved MAP kinase domain (Supplementary Fig. S2B; Agrawal *et al.*, 2003).

### Embryo development is arrested at the globular stage in *osmpk6* mutants

**Fig. 2.**
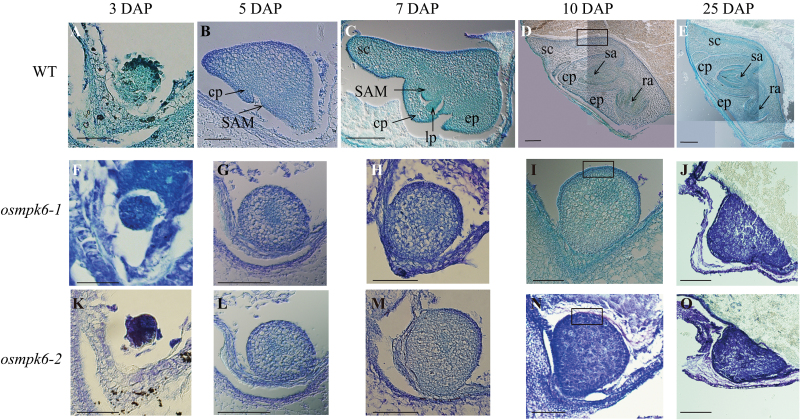
Median longitudinal sections of WT and *osmpk6* embryos at various developmental stages.

To determine when defects occur in *osmpk6* mutants, we analysed the developing embryo. At 3 DAP, the zygote formed a globular embryo in the WT (Fig. 2A). No abnormal embryos were found among the 44 seeds from the heterozygote plants (Fig. 2F, K), and all were globular, indicating that the 3 DAP mutant embryos were also normal. In WT embryos, the coleoptile primordium began to differentiate at 5 DAP, and the SAM was recognizable as a bulge protrusion at the base (Fig. 2B). Mutant embryos differed from the WT at this stage, maintaining their globular shape but still increasing in size when compared with embryos at 3 DAP. However, the coleoptile primordium and SAM were not developed in the mutant at this time (Fig. 2G, L). At 7 DAP, the primordium of the first leaf from the WT had formed below the shoot apex, and the coleoptile and SAM were elongated (Fig. 2C). The scutellum developed at the apical and dorsal ends of the embryo and the epiblast appeared opposite the scutellum. In contrast, the mutant embryos remained globular and lacked any differentiation (Fig. 2H, M). By 10 DAP, all organs had formed in the WT and embryogenesis was complete (Fig. 2D), but the seed continued to enlarge until maturation (Fig. 2E). For the mutant embryo, no further growth or differentiation was noted after 10 DAP, although the ventral side was slightly elongated (Fig. 2I, N). At 25 DAP, the dorsal side of the mutant embryo was protruding while the ventral side was flat and attached to the endosperm (Fig. 2J, O). No cellular differentiation was obvious in the mutants.

Developing embryos of WT (**A–E**), *osmpk6-1* (**F–J**), and *osmpk6-2* (**K–O**) at 3 DAP (A, F, K), 5 DAP (B, G, L), 7 DAP (C, H, M), 10 DAP (D, I, N), and 25 DAP (E, J, O). Solid boxes in D, I, and N are magnified in Fig. 6. cp, coleoptile; ep, epiblast; lp, leaf primordium; ra, radicle apex; sa, shoot apex; SAM, shoot apical meristem; sc, scutellum. Bars = 50 μm (A-C, F-H, and K-M), 100 μm (D, I, and N), or 200 μm (E, J, and O). (This figure is available in colour at *JXB* online.)

### Expression profiles of *OsMPK6*

**Fig. 3.**
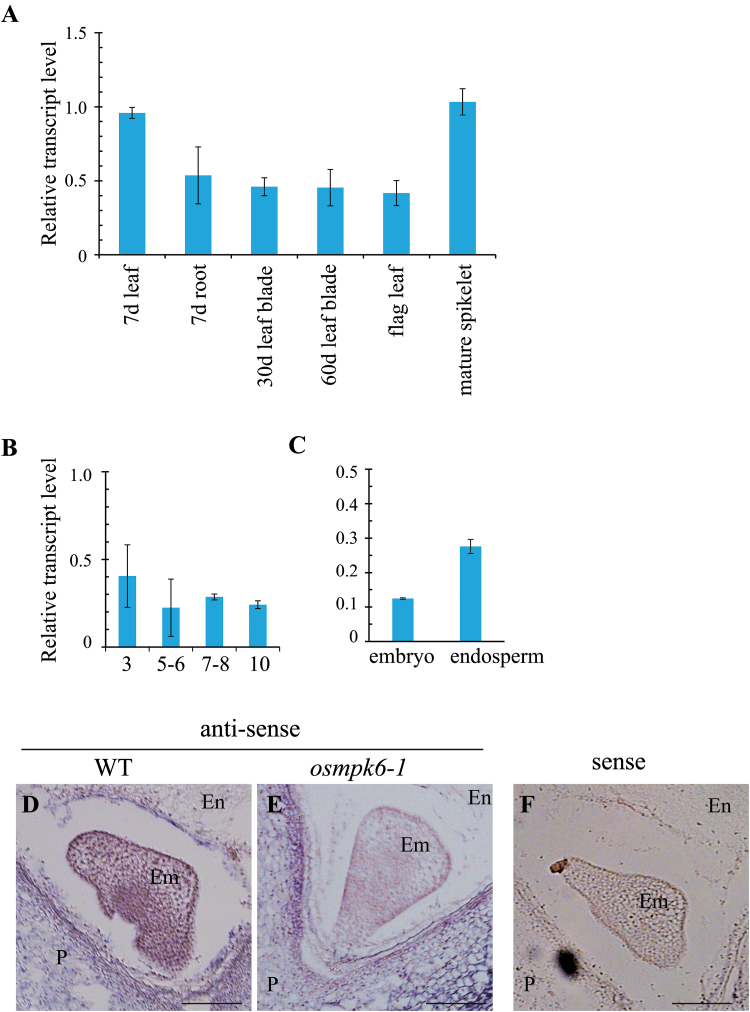
Expression profiles of *OsMPK6.*

To study the expression pattern of *OsMPK6*, we performed qRT-PCR with gene-specific primers. Transcripts were detected in all organs tested, including the leaves, roots, flag leaves, and mature spikelets (Fig. 3A). In developing seeds, expression was found at all examined developmental stages (Fig. 3B), and in both the embryo and endosperm (Fig. 3C). This analysis indicated that *OsMPK6* is ubiquitously expressed in rice.

Quantitative RT-PCR analyses of expression in various organs (**A**), developing seeds at four different stages (**B**), and embryo and endosperm from 6 DAP seeds (**C**). Transcript levels were normalized to *OsUbi1*. *In situ* hybridization with antisense *OsMPK6* in WT (**D**) and *osmpk6-1* (**E**) seeds or with sense probe as a negative control in WT seeds (**F**). Em, embryo; En, endosperm; P, pericarp. Bars = 200 μm. (This figure is available in colour at *JXB* online.)

To monitor spatial expression of this gene, we performed RNA *in situ* hybridization experiments with developing seeds at 6 DAP. Transcripts were detected in all cell types of the embryo as well as in the endosperm and pericarp (Fig. 3D). In the mutant seeds, *OsMPK6* transcripts were not present in the embryo or endosperm, whereas signals were observed in pericarp that was derived from maternal tissues (Fig. 3E). Furthermore, the sense probe that we used as a control did not detect any significant hybridization signal (Fig. 3F).

### Mutations of *OsMPK6* do not affect endosperm development

Although embryo development was affected by the mutation, seed size and colour were the same between the WT and the mutants (Supplementary Fig. S3A). Cross sections of the mature seeds showed that the pericarp and tegmen, which are maternal tissues, were normal in the mutant seeds (Supplementary Fig. S3B). The endosperm and aleurone cells were also identical between the WT and the mutants (Supplementary Fig. S3B). These findings suggest that the mutations in *OsMPK6* do not affect development of the endosperm cells.

To investigate further whether *osmpk6* endosperms are normal, we measured transcript levels of the genes involved in starch synthesis in caryopses at 10 DAP, when starch is being actively accumulated (Yamakawa *et al.*, 2007). Expression levels were similar between the WT and mutant seeds for the starch biosynthesis genes *ADP-glucose pyrophosphorylase large subunit 1* (*AGPL1*), *Starch synthase IIIa* (*SSIIIa*), and *Starch branching enzyme I* (*BEI*), and for a starch debranching enzyme, *Pullulanase* (Ohdan *et al.*, 2005; Lee *et al.*, 2015) (Supplementary Fig. S3C). These data also demonstrate that the *OsMPK6* mutations do not affect endosperm development.

### *OsMPK6* is important for the formation of the basic axis during embryogenesis

**Fig. 4.**
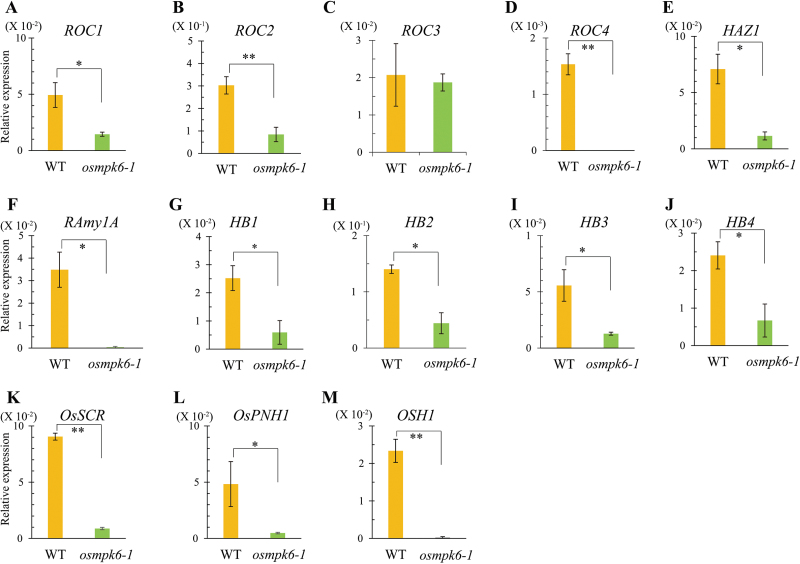
Comparisons of expression for molecular marker genes between *osmpk6-1* mutant and WT control.

To investigate them at the molecular level, we isolated *osmpk6* mutant embryos and WT embryos at 6 DAP and measured transcript levels for genes involved in embryogenesis. GLABRA2 (GL2)-type homeobox genes *Rice outermost cell-specific gene1 (ROC1*) through *ROC5* are expressed in developing embryos and show an epidermis-specific pattern of expression in the shoot and crown root apices (Ito *et al.*, 2002, 2003). The ROC proteins form homo- and heterodimers through a leucine zipper motif, and in combination they regulate epidermis differentiation. Transcript levels of *ROC1* were decreased in the *osmpk6-1* embryos (Fig. 4A), as were those of *ROC2* and *ROC4*, which are two of the three ROC genes expressed in normally developing embryos (Fig. 4B, C). By contrast, transcript levels of *ROC3* were similar between the WT and the mutant (Fig. 4D). Like *ROC1*, *HAZ1* expression is restricted to the outermost cells during early embryogenesis but is detected in the outer layer of the ventral part during later stages (Ito *et al.*, 2004). Its expression was also significantly reduced in the mutant embryos (Fig. 4E).

Quantitative RT-PCR of *ROC1* (**A**), *ROC2* (**B**), *ROC3* (**C**), *ROC4* (**D**)*, HAZ1* (**E**), *RAmy1A* (**F**), *HB1* (**G**), *HB2* (**H**), *HB3* (**I**), *HB4* (**J**), *OsSCR* (**K**), *OsPNH1* (**L**), and *OSH1* (**M**). Y-axis is the gene expression relative to *OsUbi1* transcript level. Results are averages of three independent experiments. Ten embryos were collected for each sample. Error bars show standard deviations. Statistical significance is indicated by **P < 0.01 and *P < 0.05. (This figure is available in colour at *JXB* online.)

The epithelium of the scutellum functions in transporting nutrients from the endosperm to the embryo during germination. *Rice α-amylase 1* (*RAmy1A*) is expressed on the dorsal side of that epithelium, facing the endosperm tissue (Sugimoto *et al.*, 1997). Its gene expression was also reduced in the *osmpk6-1* embryos (Fig. 4F). Transcripts of HB genes, encoding HD-ZIPIIIs, were negatively affected in mutant embryos (Fig. 4G–J). These analyses indicate that mutations in *MPK6* influence embryo development at the early globular stage, before the formation of a radial pattern.

*SCARECROW* (*SCR*) in *Arabidopsis* is specifically localized to the endodermis cell layer, which includes the cortex/endodermis initial cells that correspond to the L2 layer (Di Laurenzio *et al.* 1996). As an ortholog of *SCR* in rice, *OsSCR* is preferentially expressed in the endodermis cell layer, including the cortex (Di Laurenzio *et al.*, 1996).

A meristem-defective *Arabidopsis* mutant, *pinhead*/*zwille* (*pnh*/*zll*), has a flat meristem and fails to maintain the indeterminate state of the SAM (McConnell and Barton, 1995; Lynn *et al.*, 1999). *PNH/ZLL* is expressed in the central region of the early embryo, probably corresponding to the provascular region. The rice ortholog *OsPNH1* is also expressed in the future vascular regions of leaf primordia (Nishimura *et al.*, 2002) and has been used as an L3 vascular tissue marker (Kamiya *et al.*, 2003). Expression of these two L2 and L3 marker genes, *OsSCR* and *OsPNH1*, was decreased in *osmpk6* embryos (Fig. 4K, L).

Class 1 *KNOTTED*-like homeobox genes, such as *KNOTTED1* in maize (Smith *et al.*, 1995), *Oryza sativa homeobox 1* (*OSH1*) in rice (Sato *et al.*, 1996), and *SHOOT MERISTEMLESS* in *Arabidopsis* (Long *et al.*, 1996), are expressed in the indeterminate cells around the SAM. At the early embryo stages before cell differentiation, they are expressed in the presumptive SAM region. Therefore, *OSH1* has been used as a marker for studying the apical region in rice embryos (Sato *et al.*, 1996; Kamiya *et al.*, 2003). We noted that transcript levels of *OSH1* were also decreased in the mutant embryos (Fig. 4M). These results support previous findings that the mutants do not differentiate embryonic organs.

**Fig. 5.**
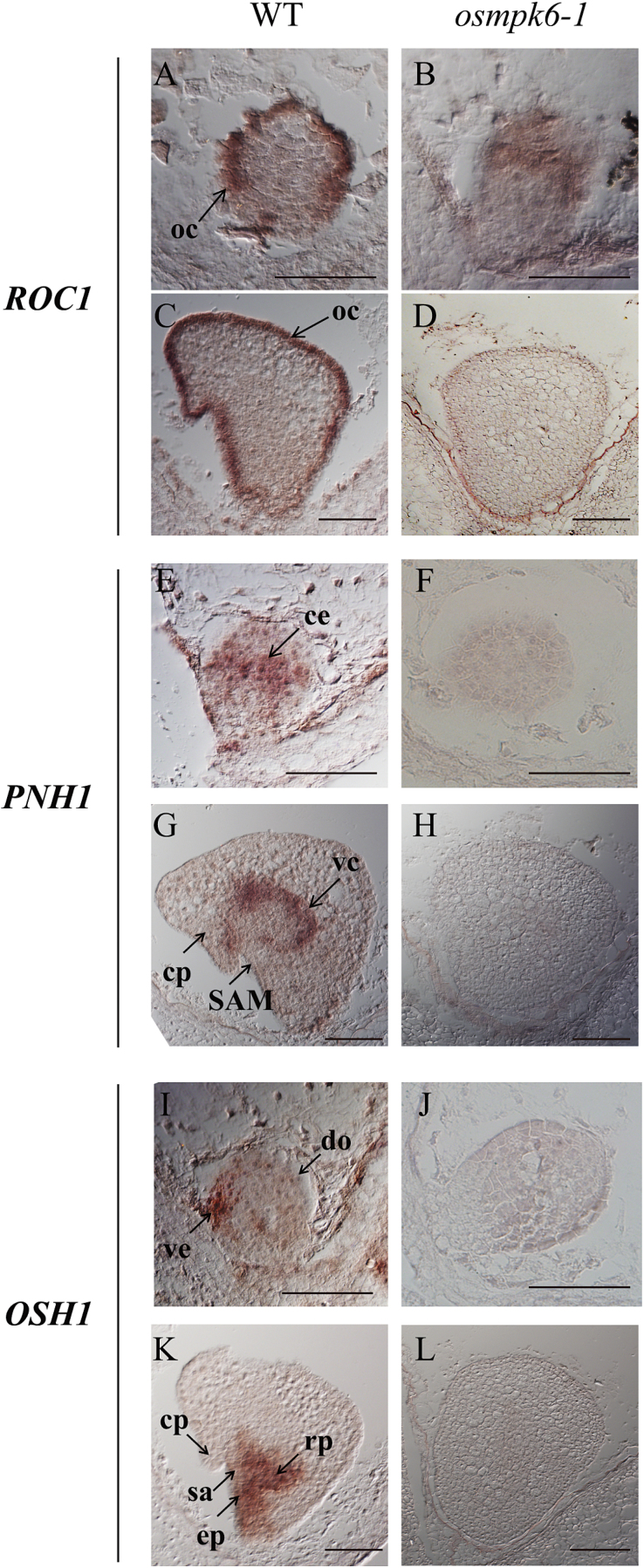
Cellular expression patterns of molecular markers from *osmpk6-1* and WT embryos.

To improve our understanding of how *osmpk6* mutations affect genes involved in basic pattern formation, we monitored the spatial expression of molecular marker genes. Because *ROC1* is specifically expressed in the L1 protoderm (Ito *et al.*, 2002), we performed RNA *in situ* hybridization experiments. In WT embryos, *ROC1* was exclusively detected in the outermost cells of globular-stage embryos at 3 DAP (Fig. 5A), when no distinct protoderm layer was yet visible. This indicates that differentiation of the L1 layer is determined shortly after fertilization. That gene was also preferentially expressed in the outermost cells of coleoptilar-stage embryos at 5 DAP, when a protoderm layer structure was evident (Fig. 5C). In *osmpk6-1* embryos, *ROC1* was weakly expressed in the outer portions (Fig. 5B, D). Transcripts of the L3 marker gene *OsPNH1* were localized in the central region at the late-globular stage (Fig. 5E) and in the vascular cylinder of coleoptilar-stage WT embryos (Fig. 5G). However, those transcripts were not detectable in the *osmpk6-1* embryos that remained at the globular stage (Fig. 5F, H).

RNA *in situ* hybridization of *ROC1* (**A–D**), *PNH1* (**E–H**), and *OSH1* (**I–L**). Wild type (A, C, E, G, I, and K) and mutant embryos (B, D, F, H, J, and L) were collected at 3 DAP (A, B, E, F, I, and J) or 5 DAP (C, D, G, H, K, and L). Bars = 50 μm (A, B, E, F, I, and J) and 100 μm (C, D, G, H, K, and L). ce, central region; cp, coleoptile; do, dorsal region; ep, epiblast; oc, outermost cell layer; rp, root primordia; sa, shoot apex; SAM, shoot apical meristem; vc, ventral cylinder; ve, ventral region (This figure is available in colour at *JXB* online.)

Expression of *OSH1*, a marker for the apical region, was restricted to a small area just below the ventral part of the globular embryo where the shoot apex arises later (Fig. 5I). In 5 DAP WT embryos, expression was observed in the shoot apex, epiblast, radicle primordia, and their intervening tissue (Fig. 5K). However, *OSH1* signals were not found in the mutants at either developmental stage (Fig. 5J, L). All of these results demonstrate that the mutant embryos failed to differentiate. Therefore, formation of the L1 layer was incomplete and embryonic organs such as the shoot apex and radicle did not develop.

**Fig. 6.**
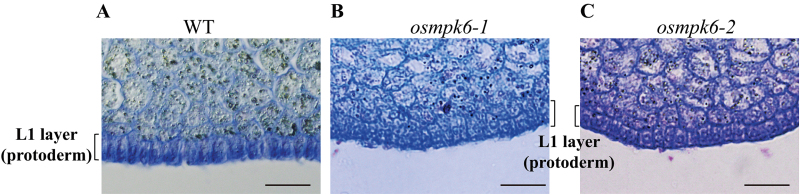
Enlarged view of L1 layer and embryo from 10 DAP seeds.

**Fig. 7.**
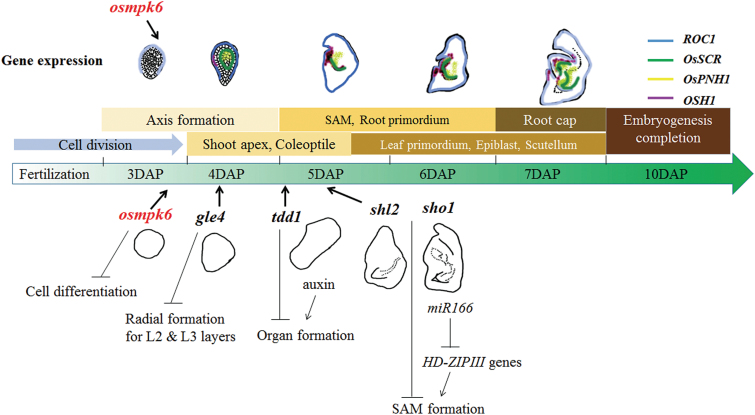
Schematic diagram of rice embryogenesis based on embryo mutants and marker genes. Genes involved in this process are presented. Arrows indicate times when mutants exhibit phenotypes. (This figure is available in colour at *JXB* online.)

During early embryogenesis, a rice embryo first establishes three axes: apical–basal, radial, and dorsal–ventral. Radial symmetry then disappears when organ differentiation begins in specific regions of the globular embryo (Ito *et al.*, 2004). This indicates that the radial axis is formed before that stage. In our *osmpk6* embryos, *ROC1* was weakly expressed at the globular stage (Fig. 5B). To examine whether protoderm differentiation had occurred in the mutant, we compared its L1 layer cells with those of the WT. In the latter embryos, the surface cells were morphologically distinguishable from the inner cells. Rectangular palisade cells were well-ordered along the outermost layer and were more intensively stained when compared with the inner cells (Fig. 6A). Palisade cells in mutant embryos were developed at the L1 layer, but were shorter than those in the WT (Fig. 6B, C). Staining intensity was similar between the L1 and inner cells, thereby suggesting that L1 cells were partially differentiated in *osmpk6* mutant embryos. Taken together, our results indicate that *OsMPK6* functions in cell differentiation during early embryo development, when the L1 radial axis is formed (Fig. 7). The gene functions prior to the development of embryonic organs such as the shoot apex and various primordia. Therefore, mutations in that gene cause down-regulation of the early-expressed *ROC1* as well as the late-expressed *OsSCR*, *OsPNH1*, and *OsH1*.

(**A**) WT, magnification of solid box in Fig. 3D. (**B**) *osmkp6-1*, magnification of solid box in Fig. 3I. (**C**) *osmkp6-2*, magnification of solid box in Fig. 3N. Bars = 20 μm. (This figure is available in colour at *JXB* online.)

### Activities of auxin and gibberellin biosynthesis genes are reduced in *osmpk6* mutants

Expression levels were examined for hormone biosynthesis genes that control embryo development. They included YUCCA genes, which are involved in auxin biosynthesis (Yamamoto *et al.*, 2007), and GA20 oxidases and GA3 oxidases that produce bioactive gibberellins (GAs) (Pearce *et al.*, 2015). Transcript levels of all five OsYUCCA genes (*OsYUCCA1*–*OSYUCCA5*) were significantly reduced in the mutant embryos (Supplementary Fig. S4A–E). Similarly, expression levels of OsGA20 oxidase genes (*OsGA20 OX-1* and *OsGA20 OX-2*) and OsGA3 oxidase genes (*OsGA3 OX-1* and *OsGA3 OX-2*) were reduced in the mutant embryos (Supplementary Fig. S4F-I). This result supports our conclusion that mutations in *OsMPK6* affect early embryo development.

### Phytoalexin biosynthesis is defective in *osmpk6* mutants

**Fig. 8.**
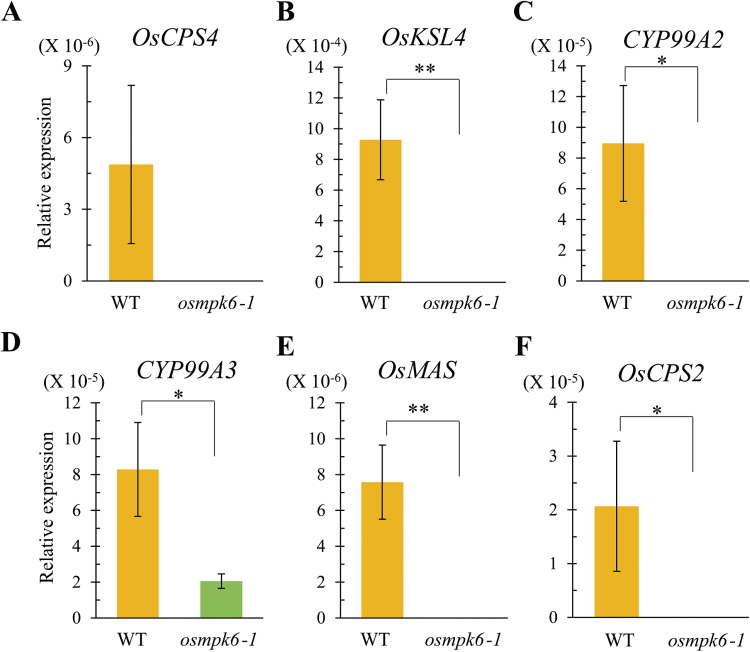
Expression profiling for phytoalexin biosynthesis genes in embryos of WT and *osmpk6-1*.

*OsMPK6* controls the biosynthesis of phytoalexins such as momilactones and phytocassanes (Kishi-Kaboshi *et al.*, 2010). Therefore, we monitored expression levels of momilactone biosynthesis genes *ent-copalyl diphosphate synthase 4* (*OsCPS4*), *Cyclase 1* (*OsCPS4*), *kaurene synthase-like gene 4* (*OsKSL4*), *CYP99A2*, *CYP99A3*, and *momilactone A synthase* (*OsMAS*). *OsCPS4* and *OsKSL4* are involved in the cyclization of geranylgeranyl diphosphate, a precursor of diterpenoid phytoalexins (Otomo *et al.*, 2004; Wilderman *et al.*, 2004). *CYP99A2* and *CYP99A3*, which encode cytochrome P450 monooxygenases, participate in the downstream oxidation steps of diterpene hydrocarbons (Wang *et al.*, 2011). *OsMAS* encodes a dehydrogenase that is also involved in momilactone biosynthesis (Shimura *et al.*, 2007). We found that expression levels of the momilactone biosynthesis genes were significantly decreased in the mutant embryos (Fig. 8A–E). In addition, we analysed transcript levels of *OsCPS2*, which is involved in the biosynthesis of phytocassanes. Its expression was also diminished in the *osmpk6-1* mutant embryos (Fig. 8F). These findings indicate that *OsMPK6* is involved in the biosynthesis of phytoalexins in developing embryos.

Quantitative RT-PCR analyses for *OsCPS4* (**A**), *OsKSL4* (**B**), *CYP99A2* (**C**), *CYP99A3* (**D**), *OsMAS* (**E**), and *OsCPS2* (**F**). Y-axis is the gene expression relative to *OsUbi1* transcript level. Results are averages of three independent experiments, using 10 embryos per sample. Error bars show standard deviations. Statistical significance is indicated by **P < 0.01 and *P < 0.05. (This figure is available in colour at *JXB* online.)

**Fig. 9.**
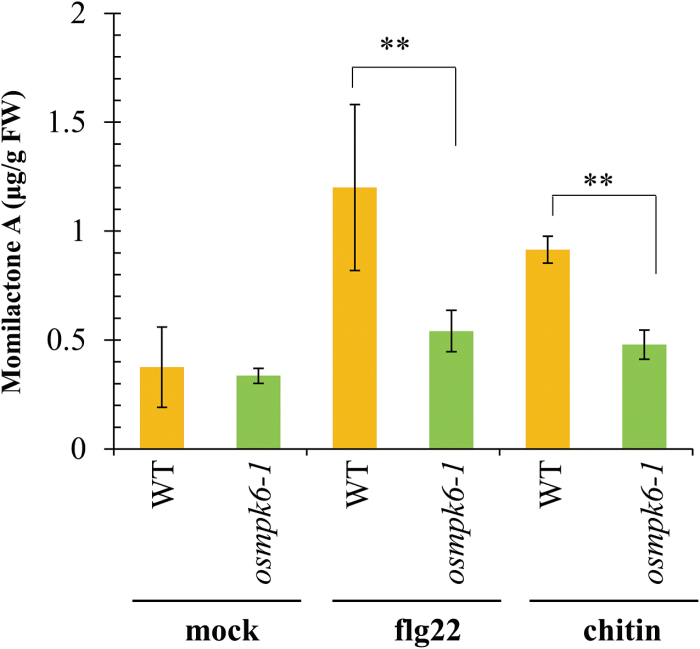
Accumulations of phytoalexin momilactone A in embryos of WT and *osmpk6-1* treated with elicitors, flg22 and chitin or water as mock control.

To verify further the biosynthetic regulation of phytoalexins by *OsMPK6*, we measured phytoalexin levels in embryos from mature seeds of the WT and mutant in response to elicitors. Treatment with flg22 and chitin induced accumulation of the major diterpenoid phytoalexin momilactone A in WT embryos, but elicitor-induced accumulation of the phytoalexin was significantly reduced in *osmpk6-1* embryos, and comparable to those of mock-treated controls (Fig. 9). This result is consistent with the decreased expression of phytoalexin biosynthesis genes found in the mutant embryos (Fig. 8).

Contents were measured by HPLC-electrospray ionization-MS/MS analysis. Results are averages of duplicated experiments. Error bars show standard deviations. Statistical significance is indicated by **P < 0.01 and *P < 0.05. (This figure is available in colour at *JXB* online.)

## Discussion

### The mutations of *OsMPK6* affect differentiation of L1 layer cells during early embryogenesis

We observed that *osmpk6* embryos did not differentiate at the globular stage and their development was arrested. However, mature mutant embryos were larger than globular WT embryos, demonstrating that cell division continued in those mutants. This *osmpk6* phenotype is distinctive from other early-embryo mutants studied in rice. In *shl* mutants, the radicle and scutellum develop normally, although the SAM is lacking (Satoh *et al.*, 2003). In *organless* mutants, epithelial cells are formed in the outermost cell layer and a cell-dense region is observed (Hong *et al.*, 1995). By comparison, no embryonic organs formed and the epithelial cells were not differentiated in the *osmpk6* mutants, such that these phenotypes were apparent at an earlier stage than in those other two mutants (Fig. 7).

Similar to our mutant embryos, the *globular embryo 4 (gle4*) mutant has globular embryos at the mature stage (Hong *et al.*, 1995). In those embryos, *ROC1* is expressed in the outermost cell layer. The palisade-like protoderm cell layer is functionally differentiated to epithelium in the *gle4* mutant, whereas those cells are not fully developed in *osmpk6* mutants. Transcripts of *RAmy1A* are also detected in *gle4* embryos but were not found in our mutants. Although both *OsSCR* and *PNH1* are expressed in *gle4*, this happens, abnormally, in the central region rather than in the ground and vascular tissues (Kamiya *et al.*, 2003). Transcripts of those genes were significantly reduced in our *osmpk6* mutants. All of these findings are evidence that the defects associated with mutations in *OsMPK6* start to occur before L1 differentiation during early embryo development and that *OsMPK6* functions before other genes that have been reported for embryogenesis in rice (Fig. 7).

### MAP kinase cascade signalling in early embryo development is conserved between *Arabidopsis* and rice

*Arabidopsis* MPK6 is involved in embryo development. Bush and Krysan (2007) reported that approximately 7% of *mpk6* seeds display burst phenotype. López-Bucio *et al* (2014) showed that the defects in embryo development caused by *mpk6* are connected to post-embryonic root architecture. The *mpk6* mutant seeds can be divided into three classes of phenotypes: bigger seeds, raisin seeds, and burst seeds. Seedlings that develop from the ‘bigger’ seeds are characterized by longer primary roots due to enhanced cell production and cell elongation in the embryos. In the other classes, mutant seedlings fail to develop primary roots, possibly as a result of an earlier defect in the division of hypophysis cells during embryo development (López-Bucio *et al.*, 2014). Moreover, the double mutant *mpk3*/*mpk6* is embryo-lethal; its zygotes do not elongate and fail to develop suspensor cells. *YODA* (*YDA*), encoding MEKK, acts upstream of *MPK3*/*MPK6*, and the basal cell lineage of *yda* mutants does not differentiate the suspensor (Lukowitz *et al.*, 2004). Mutations of *SHORT SUSPENSOR* (*SSP*), which acts upstream of *YDA*, also display a similar defect during early embryogenesis (Bayer *et al.*, 2009). These observations indicate that *SSP* functions in the YDA MAP kinase pathway to determine suspensor identity, and that the *SSP*–*YDA*–*MPK3*/*6* cascade is involved in specification of the apical–basal axis during embryogenesis (Peris *et al.*, 2010). The suspensor is the first tissue to differentiate during *Arabidopsis* embryogenesis (Kawashima and Goldberg, 2010). *OsMPK6* functions similarly in rice because *osmpk6* mutants fail to differentiate any embryonic organs. When one considers axis formation and early cell differentiation, the functional roles of *MPK6* in early embryogenesis are conserved between *Arabidopsis* and rice.

OsMPK6 is highly homologous to OsMPK3. Both are phosphorylated by OsMKK4 and interact with bHLH Rac Immunity1 protein, which functions in the pathway for innate immunity (Kim *et al.*, 2012). Because the *Arabidopsis* orthologs *MPK6* and *MPK3* are functionally redundant (Hamel *et al.*, 2006), *OsMPK3* may also be redundant to *OsMPK6*. We observed embryo-lethal phenotypes from single *osmpk6* mutants that were probably due to gene transcript levels in the developing seeds. We also showed that *OsMPK6* is highly expressed in developing embryos. However, *OsMPK3* is only weakly expressed in developing embryos but strongly expressed in the endosperm (NCBI Gene Expression Omnibus; GSE11966 http://www.ncbi.nlm.nih.gov/geo/query/acc.cgi?acc=GSE11966, Xue *et al.*, 2009). Therefore, the lack of abnormal phenotypes in *ospmk6* endosperms likely results from the strong expression of *OsMPK3* balancing the deficiency of *OsMPK6*.

### OsMPK6 is required for activities of auxin and GA biosynthesis genes

Expression levels of auxin biosynthesis genes were significantly decreased in the defective embryos of *osmpk6*. In *Arabidopsis*, *YUC1* and *YUC4* are specifically expressed in apical protodermal cells from the globular stage (Robert *et al.*, 2013). PIN-FORMED (PIN) auxin transporters participate in apical–basal axis formation by regulating auxin gradients (Friml *et al.*, 2003; Jenik and Barton, 2005). The embryos from *Arabidopsis pin7* mutants lack polarity in their cell files and have no recognizable proembryos (Friml *et al.*, 2003). In a rice *tdd1* mutant that shows an organless embryo phenotype, the amount of auxin is significantly reduced (Sazuka *et al.*, 2009). Embryogenesis is controlled by auxin distribution in *Triticum aestivum* and maize (Fischer-Iglesias *et al.*, 2001; Forestan *et al.*, 2010). These previous reports together with our observations indicate that proper auxin levels are needed for normal embryonic organ formation and that OsMPK6 may be involved in auxin biosynthesis during embryogenesis.

GA biosynthesis genes were also affected in *osmpk6* mutants. During the globular and heart stages in *Arabidopsis*, this hormone is quickly accumulated in the seeds (Locascio *et al.*, 2014). In *Brassica napus*, GA is required for cell elongation in the axis of microspore-derived embryos (Hays *et al.*, 2002). Findings from all of these studies suggest that GA promotes cell growth and expansion during embryo differentiation.

### Phytoalexin biosynthesis, directed by OsMKK4-OsMPK6, is a putative signal for cell differentiation

The MPK3/MPK6 cascade is essential for the biosynthesis of phytoalexins. These antimicrobial compounds accumulate rapidly at sites of infection to inhibit the growth of fungi and bacteria (Tsuji *et al.*, 1992). They also play an important role in somatic embryogenesis (Kouakou *et al.*, 2008). Trans-resveratrol, a phytoalexin, is absent in suspension cells of cotton (*Gossypium hirsutum* L.) cultivar R405-2000, which is unable to produce somatic embryos. By contrast, trans-resveratrol accumulates in cell suspensions of the embryogenic Coker 312 when embryogenic structures are induced (Kouakou *et al.*, 2008). Furthermore, medicarpin (3-hydroxy-9-methoxypterocarpan), one of the major phytoalexins produced by dietary legumes, stimulates osteoblast differentiation *in vitro* (Bhargavan *et al.*, 2012). Therefore, phytoalexins appear to function as a signal for cell differentiation. Both momilactones and phytocassanes accumulate in WT cells after elicitor treatment, but their levels are significantly reduced in *osmpk6* mutant cells (Kishi-Kaboshi *et al.*, 2010). We also found that the level of momilactone A was reduced in *osmpk6* mutant embryos (Fig. 9). This suggests that the lack of phytoalexins negatively affects embryo development and that the embryo-lethal phenotype of *osmpk6* is possibly due to an insufficient amount of phytoalexins in those mutant embryos. Further studies are needed to clarify the role(s) of these compounds during embryogenesis.

## Supplementary data

Supplementary data are available at *JXB* online.

Fig. S1. Characterization of *OsMPK6* activation tagging line.

Fig. S2. Alignments of MPK family proteins.

Fig. S3. Analysis of endosperm development in WT and *osmpk6*.

Fig. S4. Expression profiling of biosynthesis genes for auxin and gibberellin in embryos of WT and *osmpk6-1*.

Table S1. Primer sequences used in this study.

Supplementary Data
