# Supplementary material for: OsMPK6 plays a critical role in cell differentiation during early embryogenesis in Oryza sativa
Source: J Exp Bot. 2016 Feb 24;67(8):2425–37. doi: 10.1093/jxb/erw052 (PMC4809295; doi:10.1093/jxb/erw052)
Supplement: Supplementary Data [file supp_67_8_2425__index.html]

 OsMPK6 plays a critical role in cell differentiation during early embryogenesis in Oryza sativa — OsMPK6 plays a critical role in cell differentiation during early embryogenesis in Oryza sativa — Supplementary Data 

# *OsMPK6* plays a critical role in cell differentiation during early embryogenesis in *Oryza sativa*

## Supplementary Data

Data files

- supplementary\_figures\_S1\_S4\_table\_S1.pdf - Supplementary Data
