# Supplementary material for: OsMPK6 plays a critical role in cell differentiation during early embryogenesis in Oryza sativa
Source: J Exp Bot. 2016 Feb 24;67(8):2425–37. doi: 10.1093/jxb/erw052 (PMC4809295; doi:10.1093/jxb/erw052)
Supplement: Supplementary Data [file supp_erw052_supplementary_figures_S1_S4_table_S1.pdf]

Supplementary material

OsMPK6 plays a critical role in cell differentiation during early embryogenesis in rice

Jakyung Yi†, Yang-Seok Lee†, Dong-Yeon Lee, Man-Ho Cho, Jong-Seong Jeon and Gynheung An*

Supplementary Fig. S1. Characterization of *OsMPK6* activation tagging line.

(A) In Line 2A-10648, T-DNA was inserted 2,124 bp downstream of *OsMPK6* stop codon. Dark-gray boxes indicate exons; light-gray boxes, UTRs; lines connecting boxes, introns. Arrows a and b are primers used for analysis of *OsMPK6* transcript levels. Arrows c, d, and LB indicate primers used for genotyping the line. Scale bar = 1 kb.
(B) Quantitative RT-PCR analyses of *OsMPK6* in WT and *OsMPK6-D* embryos. Samples were harvested after imbibition. Y-axis, gene expression relative to *OsUbi1* transcript level. Error bars show standard deviations. n = 2 (DJ) or 4 (*OsMPK6-D*). Statistical significance is indicated by * ($P < 0.05$).
(C) Embryo phenotypes of WT and *OsMPK6-D*. Bars = 1 mm.

A

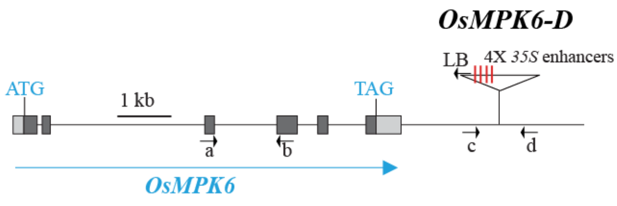

B

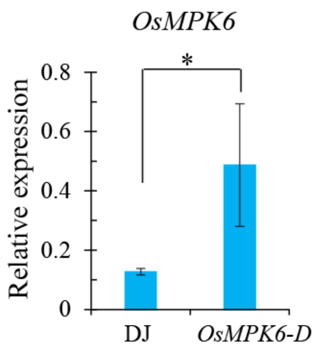

C

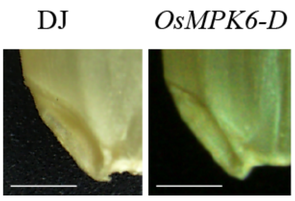

Supplementary Fig. S2. Alignments of MPK family proteins.

(A) Phylogenetic analysis of MPK family proteins from rice and *Arabidopsis*, obtained by MEGA version 6.0 via the NJ method with 1000 replicates; the handling gap option was pair-wise deletion. Scale bar corresponds to 0.05 amino acid substitutions per residue. (B) Multiple alignment of TEY group MPK proteins, performed by Bioedit program (version 7.2.5). Identical amino acids are shaded in black; similar amino acids, in gray. Blue asterisk (*) shows MAPK domain (PS01351; F-x(10)-R-E-x(72,86)-R-D-x-K-x(9)-[CS]). PK subdomains are indicated by Roman numeral (I to XI) at top of each row; aa positions, by pink line; phosphorylation-activation motif (TEY), by yellow box; docking groove for binding substrates, by green dashed line. This domain includes (V/A/T/S)vELMDTDLHQII(R/K)SNQxL(S/T)x(D/E)Hcx(V/F)F around sub-domain V and (L/V/I)NANCDL, between sub-domains VI and VII.

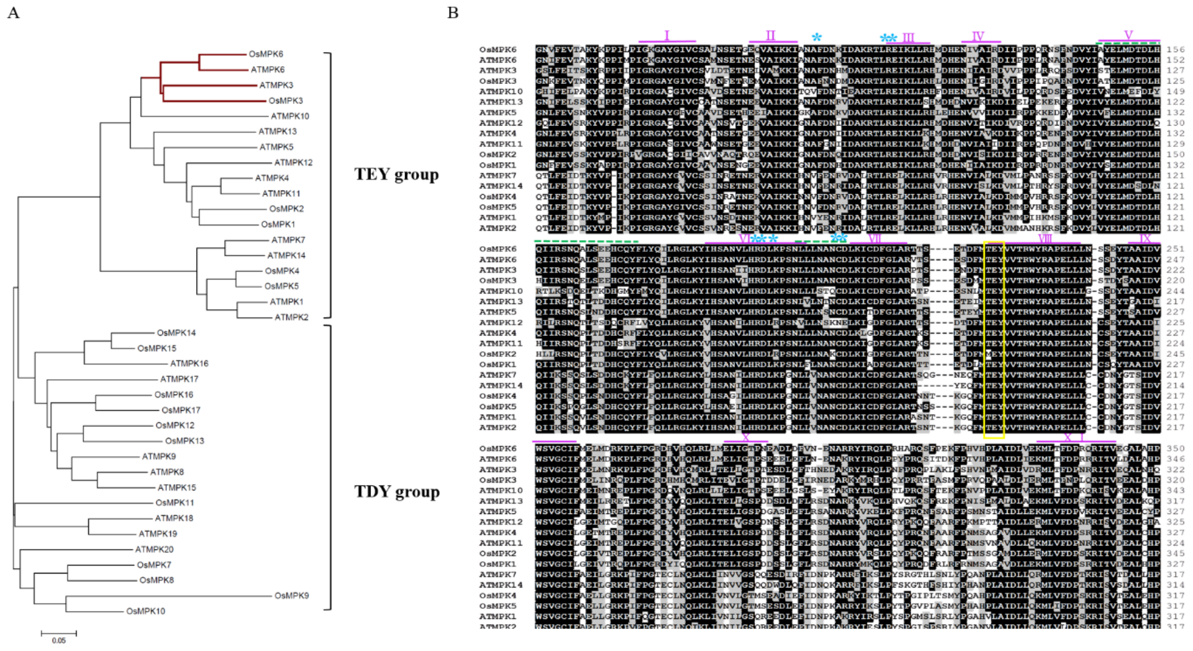

Supplementary Fig. S3. Analysis of endosperm development in WT and *osmpk6*.

(A) Mature seeds of WT, *osmpk6-1*, and *osmpk6-2*. Arrows indicate embryos. White boxes show ventral regions that are sectioned in B. Bars = 1 mm. (B) Peripheral regions of WT, *osmpk6-1*, and *osmpk6-2*. AL, aleurone layer; En, endosperm; P, pericarp; T, tegmen. Bars = 50 µm. (C) Expression levels of genes involved in starch biosynthesis, obtained via qRT-PCR analyses of *AGPL1*, *SSIIa*, *BE1*, and *PUL* from 7 DAP seeds of WT and *osmpk6-1*. Y-axis, relative transcript level compared with that of *OsUbi1*. Error bars indicate standard deviations; *n* = 4.

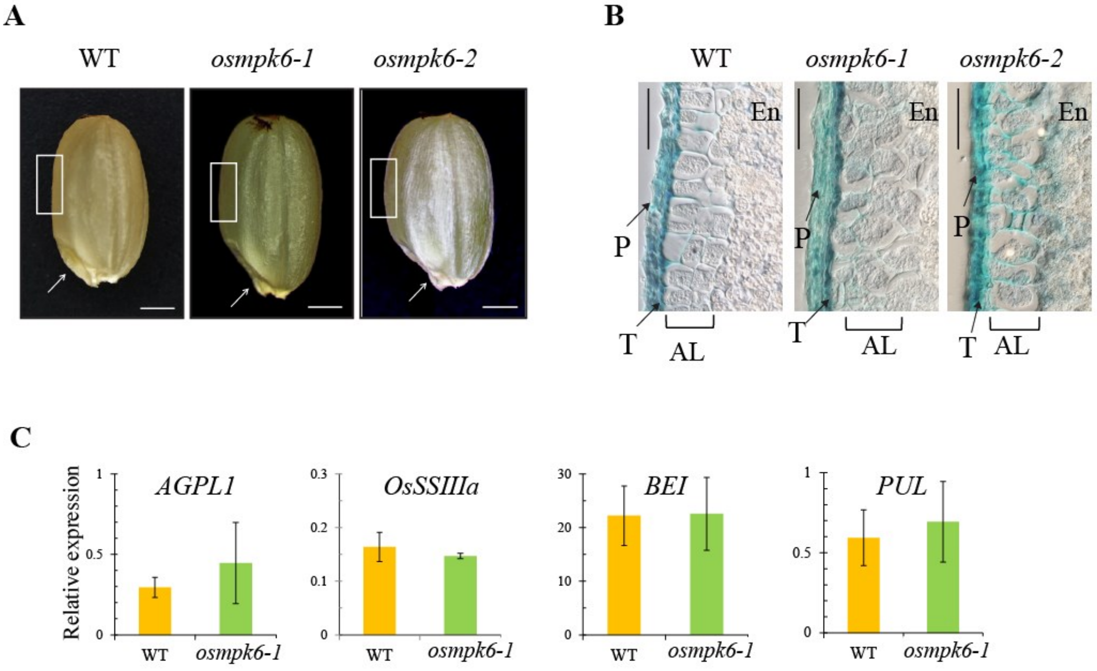

Supplementary Fig. S4. Expression profiling of biosynthesis genes for auxin and gibberellin in embryos of WT and *osmpk6-1*.

Quantitative RT-PCR of *OsYUCCA1* (A), *OsYUCCA2* (B), *OsYUCCA3* (C), *OsYUCCA4* (D), *OsYUCCA5* (E), *OsGA20 OX-1* (F), *OsGA20 OX-2* (G), *OsGA3 OX-1* (H), and *OsGA3 OX-2* (I) in *osmpk6-1* and WT control. Y-axis, gene expression relative to *OsUbi1* transcript level. Results are averages of three independent experiments. Ten embryos were collected for each sample. Error bars show standard deviations. Statistical significance is indicated by ** (P < 0.01) and * (P < 0.05).

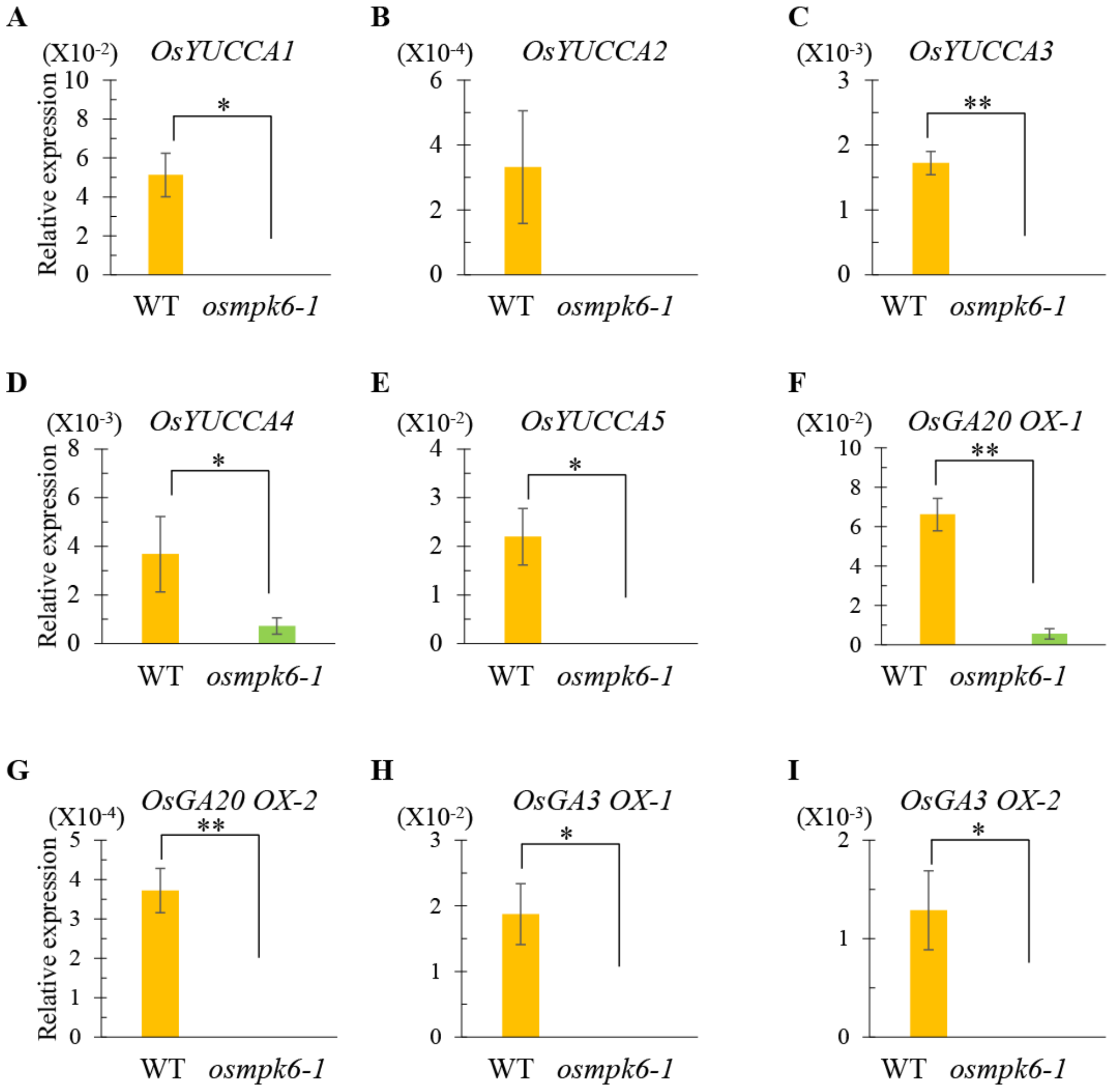

Supplementary Table S1. Primer sequences used in this study.

Primer name	purpose	Sequence (5′-3′)
f		CAACTTCCTAGACATGCAAG
r		TTGATCTGCTTCTGCTCCAGC
c	genotyping	CCATTTCTTCAAGGGCTATC
d		GAACGATGTGAGGGCTGCAT
LB		CTAGAGTCGAGAATTCAGTACA
a		GATGGATACTGATCTGCATC
b		GTTGCTGGGCTTCAAGTCTC
AGPL1 qF		GGAAGACGGATGATCGAGAAAG
AGPL1 qR		CACATGAGATGCACCAACGA
OsSSIIIa qF		GCCTGCCCTGGACTACATTG
OsSSIIIa qR		GCAAACATATGTACACGGTTCTGG
BEI qF		TGGCCATGGAAGAGTTGGC
BEI qR		CAGAAGCAACTGCTCCACC
PUL qF		GCTGTCGCTTCTTATGATGCTC
PUL qR		AAGTGGTCCAGTATAAGCAAACAT
ROC2 qF		ATCAGTGGGAAGTCAAGAAC
ROC2 qR		GAGAGGCGAACATGAAGAGG
ROC3 qF		GAGCAACATGGGATCAGACAC
ROC3 qR		TGAGTAGCTGTTAGTGTTGG
ROC4 qF		CTGCTACATCTGTGTGGATG
ROC4 qR		CAGGAGATAAGGTTGCTCAC
HAZ1 qF		GAAGACTGGCAGGTGAAACTC
HAZ1 qR		GTCCTAATTCAAGGTAGTACAG
RAmy1A qF		CAAAGATTGGACCAAGATACG
RAmy1A qR		GAAGTACTTCGTGGACAATTG
HB1 qF		GTGCAGGCAAGGAGATAAGAG
HB1 qR	qRT-PCR	CTGAAGCCGTGAAGACTCCTTC
HB2 qF		TGCTCGCAGTCATCGTCGTTG
HB2 qR		CCAGATCAAATTAGTGCAAAAC
HB3 qF		GGACCCCAGATCGGAATGA
HB3 qR		AAGTGTGCGTGTCGCAGATG
HB4 qF		CAGGCTTCCGTGTGATACCA
HB4 qR		AAGTGTGCGTGTCGCAGATG
OsSCR qF		CGATGGATACACGCTTATTGAG
OsSCR qR		GATCAAGTGATACTTCAGCTC
OsYUCCA1 qF		TCATCGGACGCCCTCAACGTCGC
OsYUCCA1 qR		GGCAGAGCAAGATTATCAGTC
OsYUCCA2 qF		GTCCAAAGGGAGGAGTCGTCCAG
OsYUCCA2 qR		GCATGATGTTTACACCCGGCCTT
OsYUCCA3 qF		GTGAGAACGGGCTCTACTCGGTCG
OsYUCCA3 qR		GCTTATGCATGACCGATGAACACG
OsYUCCA4 qF		GCAGAATGGCCTGTACGCTGTTGG
OsYUCCA4 qR		CAGACCAGCACATGACGTGTCTAC
OsYUCCA5 qF		ACCTCCTACGACGCCGCCATGATC
OsYUCCA5 qR		CTCCCAACACAGCGACGACAGAAC
OsGA20 OX-1 qF		TACGGGCCGACATGCGCACG
OsGA20 OX-1 qR		GCATGCATGTAGGAGTAGCTAGG
OsGA20 OX-2 qF		GCGCCATGGTCATCAACATCGG

OsGA20 OX-2 qR		AGCGCATGAGGTCGGCCCAGGT
OsGA3 OX-1 qF		ATGGAGGAGTACGACTCGTCGTCGATGAGAG
OsGA3 OX-1 qR		CTCTGCAGGATGAAGGTGAAGAAGCCTG
OsGA3 OX-2 qF		TCTCCAAGCTCATGTGGTCCGAGGGCTA
OsGA3 OX-2 qR		TGGAGCACGAAGGTGAAGAAGCCCGAGT
OsCPS4-F		TGACGAGGCTGGGCATATC
OsCPS4-R		TCTGGAGTCCAGTTCCTGAAA
OsKSL4-F		CGCCTTTGTAACTCTAAGGTA
OsKSL4-R		ACGTAAAAGGCTTGTATATC
OsMAS-F		AAATGATTTGGGACCAGTGG
OsMAS-R		GACAGAATCTAGCTAGCGATGGA
CYP99A2-F		ATACGGCTCCTACCCAAAGC
CYP99A2-R		CATTATTCCGGGGACAAACAT
CYP99A3-F		TCGCTTACGTGCTTGCATAC
CYP99A3-R		CAAAGCACGGGGTATCAACT
CPS2-QPCR-F		CGAGGAGCTTACTGTACGC
CPS2-QPCR-R		TGAGCAGATCTCGATTGTG
OsUBQ1 qF		TGAAGACCCTGACTGGGAAG
OsUBQ1 qR		CACGGTTCAACAACATCCAG
MPK6 F2	in situ	GTCCATCAATTACGTCTACTA
MPK6 R2		CTGGTAATCAGGGTTGAAC
ROC1 F	qRT-PCR / in situ	ACAGCAACCCTCAGTAGTAG
ROC1 R		CCAACAAGCAACAACCACAAGT
OsPNH1 F		CCACTGGGACGAACGGAAC
OsPNH1 R		GATACAACAACTATTATACATGC
OSH1 F		ACGAGATGCAGTTCGTGATGATG
OSH1 R		TCGAACGATCAGCAAATTATATAATC
